# Novel Approaches for the Treatment of Maxillofacial Defects

**DOI:** 10.3390/bioengineering11100995

**Published:** 2024-10-02

**Authors:** Mina Medojevic, Aleksandar Jakovljevic, Raphaël Devillard, Olivia Kérourédan

**Affiliations:** 1 BioTis, U1026, Institut National de la Santé et de la Recherche Médicale, Université de Bordeaux, F-33000 Bordeaux, France; mina.medojevic@gmail.com (M.M.); raphael.devillard@u-bordeaux.fr (R.D.); 2 Department of Pathophysiology, School of Dental Medicine, University of Belgrade, 11000 Belgrade, Serbia; a.jakovljevic@stomf.bg.ac.rs; 3 Implant-Research Center, School of Dental Medicine, University of Belgrade, 11000 Belgrade, Serbia; 4 UFR des Sciences Odontologiques, Université de Bordeaux, F-33076 Bordeaux, France; 5 CHU de Bordeaux, Pôle de Médecine et Chirurgie Bucco-Dentaire, F-33000 Bordeaux, France; 6 Centre de Compétence des Maladies Rares Orales et Dentaires (O-Rares), CHU de Bordeaux, F-33000 Bordeaux, France; 7 Centre de Compétence des Maladies Osseuses Constitutionnelles (MOC), CHU de Bordeaux, F-33000 Bordeaux, France

Maxillofacial defects, located in a region characterized by a complex interplay of soft and hard tissues, along with a sophisticated capillary and neural network, have long posed significant challenges in both clinical practice and research. These defects, resulting from trauma, tumors, congenital anomalies, or other conditions, have a profound impact on patients’ psychological and physiological well-being [1]. The need for effective treatments that restore both form and function is significant. Despite progress in the field, the management of these defects remains challenging, particularly due to their diverse etiology and the intricate anatomical structures involved [2].

Current treatments typically involve the use of autologous bone grafts for hard tissue reconstruction and prosthodontic devices or sutures for soft tissue management [3]. While these gold standard approaches offer reliable outcomes, they are often associated with significant limitations. The use of autologous bone grafts, for instance, involves risks related to donor site morbidity, limited availability of bone material, and variable long-term success [4]. Similarly, traditional prostheses and suturing techniques may lead to esthetic and functional shortcomings, which can have a lasting impact on the patient’s quality of life [5].

Consequently, there is a growing interest in the development of novel approaches that leverage biomaterials, tissue engineering, and regenerative medicine to improve patient outcomes [6,7,8].

In this Special Issue of Bioengineering, we have curated seven papers that explore innovative strategies aimed at addressing the limitations of traditional treatments for maxillofacial defects. These studies highlight significant advancements in bone and soft tissue regeneration, the use of biomaterials, and the application of cutting-edge technologies such as laser-assisted fabrication and bioprinting. Below, we summarize the key findings of the included articles, discuss gaps in the current knowledge, and outline potential directions for future research.

A promising direction in maxillofacial reconstruction is the use of biomaterials combined with stem cells to promote bone regeneration. In their study, Destrez et al. [9] explored the use of a granular bone substitute enriched with mesenchymal stem cells (MSCs) for the treatment of alveolar cleft defects. Alveolar clefts are particularly challenging to treat, as they often require multiple interventions over the course of a patient’s life. The authors developed an optimized in vivo model using rats, in which they assessed the bone regeneration potential of synthetic biphasic calcium granules combined with MSCs. The results were encouraging, demonstrating significant increases in both bone volume and mineral density in the experimental groups compared with controls. However, the study also revealed that there was no significant difference between the groups treated with biomaterials alone and those treated with biomaterials combined with MSCs. This finding underscores the need for further research into optimizing stem cell integration within biomaterial scaffolds, as the quantity and viability of the stem cells may play a critical role in their effectiveness. This study represents an important step forward in the development of alternatives to autologous bone grafts. However, the lack of a marked difference between the biomaterial-only and stem-cell-enhanced groups highlights an ongoing challenge in the field: optimizing the interaction between biomaterials and stem cells to achieve consistent and reliable outcomes in bone regeneration. Future research should focus on improving the delivery and retention of stem cells within the defect site, as well as exploring ways to enhance their osteogenic potential.

In the second article, Elahi et al. [10] performed a study investigating the effect of different sintering temperatures on the mechanical and biological properties of bovine bone grafts. Bovine bone has been widely used as a substitute for human bone in grafting procedures due to its availability and biocompatibility. However, the processing of bovine bone for clinical use, particularly the sintering process, can significantly impact its structural and mechanical characteristics. The study compared bone blocks subjected to various thermal treatments, ranging from boiling to sintering at high temperatures (550 °C and 1100 °C). The authors found that while higher sintering temperatures increased the crystallinity and compressive strength of the grafts, they also led to the formation of microcracks and a reduction in the organic content of the bone. These microstructural changes, although beneficial in terms of mechanical strength, could potentially compromise the graft’s long-term durability and integration with the host tissue. These findings suggest that while high-temperature sintering improves certain aspects of bone graft performance, there is a trade-off between mechanical strength and biological compatibility. Future research should explore alternative processing methods that can maintain the desirable mechanical properties of the graft without compromising its biological functionality. Additionally, the introduction of bioactive coatings or surface modifications could help mitigate the negative effects of high-temperature sintering and enhance the integration of the graft with the surrounding bone.

In another innovative approach to bone regeneration, Touya et al. [11] explored the use of laser-assisted surface patterning to influence cell organization and enhance the osteoinductive properties of bone grafts. By using a high-precision laser to create microgrooves on the surface of the cortical bone, the researchers were able to guide the orientation and organization of cells seeded onto the graft. The study demonstrated that specific hatch distances created by the laser patterning influenced the cell alignment, with certain patterns promoting more organized and functional cell structures. This technique holds great promise for improving the integration and functionality of bone grafts, as the ability to direct cell organization could lead to more efficient bone regeneration and faster healing times. Further investigations in this area should focus on optimizing laser parameters to achieve the best possible cell organization and exploring the potential for combining laser-assisted patterning with other regenerative technologies, such as growth factor delivery or stem cell seeding. Additionally, further studies are needed to investigate the long-term effects of this technique on graft integration and bone remodeling in vivo.

Two reviews were included in this Special Issue, also aiming to provide insights in the field of bone tissue engineering.

In the first review, Touya et al. [12] examined the potential of silica-based inks for use in 3D printing applications in bone tissue engineering. Silica has been shown to enhance the bioactivity of biomaterials, particularly in promoting bone formation and remodeling. The review identified several promising formulations of silica-derived inks that have demonstrated efficacy in preclinical models of bone regeneration. However, the review also highlighted significant variability in the experimental methods and outcomes reported in the literature. This lack of standardization makes it difficult to draw definitive conclusions about the best formulations and approaches for clinical use. The authors emphasized the need for more rigorous and standardized protocols in future research, as well as the importance of translating these findings from small animal models to human clinical trials.

In the second review, Chen and Huang [13] reviewed the use of nanomaterials in periodontal tissue engineering, focusing on their potential to improve the regeneration of alveolar bone and periodontal ligaments. Periodontitis, a common condition characterized by the destruction of these structures, presents a significant clinical challenge. Traditional treatments, such as guided tissue regeneration, are effective but often fail to fully restore the functional integration of soft and hard tissues. Nanomaterials offer several advantages in this context, including enhanced biocompatibility, antibacterial properties, and the ability to promote cellular adhesion and differentiation. The authors discussed various types of nanomaterials that are currently under investigation, including nanofibers, nanoparticles, and nanocomposites. While the results of preclinical studies are promising, concerns about the long-term safety and environmental impact of nanomaterial production remain. Future research should focus on developing more sustainable methods for synthesizing nanomaterials and on conducting long-term studies to assess their safety and efficacy in clinical applications. Additionally, the integration of nanomaterials with advanced fabrication techniques, such as 3D bioprinting, could further enhance their potential for use in regenerative dentistry.

In parallel, another study conducted by Gerasimenko et al. [14] explored the use of laser soldering technology for the reconstruction of soft tissues. Traditional suturing methods, while effective, often lead to scarring and delayed healing. In contrast, laser soldering, which uses a combination of albumin-based solders and a temperature-controlled diode laser, was shown to promote faster wound closure and reduce scarring in animal models. The study demonstrated that laser-soldered wounds healed with minimal inflammation and fibrous tissue formation, resulting in improved esthetic outcomes. The use of laser soldering could represent a significant advancement in soft tissue reconstruction, particularly in facial and maxillofacial surgery, where minimizing scarring is of paramount importance. In the future, this technology could be refined and used in more complex surgical procedures, such as laparoscopic and robotic-assisted surgeries. Additionally, further studies should focus on optimizing the composition of the solders to improve their strength and biocompatibility.

Finally, in the field of conservative medicine, there have also been efforts to further improve the early detection of dental caries in order to prevent upcoming complications arising from late diagnostics. In their study, Welch et al. [15] introduced a novel imaging technique for the early detection of dental caries using mid-infrared thermophotonic imaging. The current methods for detecting dental caries are often limited to advanced lesions, with early-stage lesions going undetected until they cause significant damage. Thermophotonic imaging, which takes advantage of the differences in the optical and thermal properties between sound and carious enamel, offers a non-invasive and highly sensitive method for identifying early-stage caries.

The authors revealed that thermophotonic imaging could detect demineralization in extracted teeth at very early stages, potentially allowing for earlier intervention and the use of remineralization therapies to prevent further damage. While the technique is still in its experimental stages, it holds great promise for improving the early diagnosis of dental caries and reducing the need for more invasive treatments.

The future of maxillofacial defect treatment lies in the continued integration of advanced technologies with regenerative medicine. Personalized approaches, such as 3D bioprinting and patient-specific grafts, hold great promise for improving the outcomes of reconstructive surgeries. Additionally, the use of bioactive materials, combined with growth factors and stem cells, could lead to more effective and predictable tissue regeneration. Emerging imaging technologies, such as thermophotonic imaging and advanced tomography techniques, will also play a critical role in the early diagnosis and treatment planning for maxillofacial defects. These technologies have the potential to revolutionize the way clinicians approach both hard and soft tissue reconstruction, allowing for more precise and minimally invasive interventions.

In conclusion, the papers presented in this Special Issue offer valuable insights into the future of maxillofacial defect treatment. By highlighting both the progress and challenges in this rapidly evolving field, we hope to inspire further research that will ultimately improve patient outcomes and advance clinical practice. The innovative approaches discussed here will undoubtedly serve as a foundation for future breakthroughs in tissue engineering, regenerative medicine, and reconstructive surgery.
